# Effectiveness of Deep Cervical Fascial Manipulation^®^ and Sequential Yoga Poses on Pain and Function in Individuals with Mechanical Neck Pain: A Randomised Controlled Trial

**DOI:** 10.3390/life13112173

**Published:** 2023-11-06

**Authors:** Prabu Raja G, Shyamasunder Bhat, Ranganath Gangavelli, Anupama Prabhu, Antonio Stecco, Carmelo Pirri, Vennila Jaganathan, César Fernández-de-Las-Peñas

**Affiliations:** 1 Department of Exercise and Sports Science, Manipal College of Health Professions, Manipal Academy of Higher Education (MAHE), Manipal 576104, India; prabu.raja@manipal.edu; 2 Department of Orthopedics, Kasturba Medical College, Manipal Academy of Higher Education (MAHE), Manipal 576104, India; 3 Department of Physiotherapy, GITAM School of Physiotherapy, GITAM University, Visakhapatnam 530045, India; rgangave@gitam.edu; 4 Department of Physiotherapy, Manipal College of Health Professions, Manipal Academy of Higher Education (MAHE), Manipal 576104, India; anupama.prabhu@manipal.edu; 5 Department of Rehabilitation Medicine, New York University, New York, NY 10012, USA; antonio.stecco@nyulangone.org; 6 Department of Neurosciences, Institute of Human Anatomy, University of Padua, 35121 Padova, Italy; carmelo.pirri@unipd.it; 7 Department of Statistics, Manipal College of Health Professions, Manipal Academy of Higher Education (MAHE), Manipal 576104, India; vennila.j@manipal.edu; 8 Department of Physical Therapy, Occupational Therapy, Rehabilitation and Physical Medicine, Universidad Rey Juan Carlos, 28008 Madrid, Spain; cesar.fernandez@urj.es

**Keywords:** cervical pain, musculoskeletal manipulation, stretching, soft tissue mobilisation

## Abstract

Background: This study aimed to investigate the effect of fascial manipulation (FM) of the deep cervical fascia (DCF) and sequential yoga poses (SYP) on pain and function in individuals with mechanical neck pain (MNP). Method: Following the predefined criteria, ninety-nine individuals with MNP were recruited, randomised, and assigned to either the intervention group (IG) (n = 51) or the control group (CG) (n = 48). Individuals in the IG received FM (4 sessions in 4 weeks) and the home-based SYP (4 weeks). The CG participants received their usual care (cervical mobilisation and thoracic manipulation (4 sessions in 4 weeks) along with unsupervised therapeutic exercises (4 weeks). The participants underwent baseline and weekly follow-up measurements of pain using a numerical pain rating scale (NPRS) and elbow extension range of motion (EEROM) during the upper limb neurodynamic test 1 (ULNT1). The baseline and the fourth session follow-up measurements of the patient-specific functional scale (PSFS) and fear-avoidance behavior Questionnaire (FABQ) were also taken. Results: A repeated-measures ANOVA was performed. There were statistically significant differences between the IG and CG on the NPRS third and fourth sessions, with mean differences (MD) of −1.009 (*p* < 0.05) and −1.701 (*p* < 0.001), respectively. Regarding EEROM, there was a 20.120° difference (*p* < 0.001) in the fourth session between the groups. The MD in FABQ was −5.036 (*p* < 0.001), but there were no significant differences in PSFS between the groups during the follow-up. Conclusion: FM and SYP can aid in reducing pain and fear-avoidance behaviour and improve the function and extensibility of the upper quarter region.

## 1. Introduction

A common musculoskeletal condition in adults, mechanical neck pain (MNP) is characterised by non-specific discomfort at the cervicothoracic junction that may or may not radiate to the upper extremity (UE) [1,2,3]. The possibility of persistent symptoms in nearly half of MNP patients significantly increases overall disability and burdens society tremendously [1,3]. A study on the causes of upper quadrant pain revealed that adults’ neck pain increased with prolonged computer and mobile device use without physical activity [4]. About two-thirds of people experience non-specific neck pain at some point, especially in middle age. It is primarily caused by postural or mechanical issues [5].

MNP and its associated symptoms are commonly treated conservatively with electrical modalities, manual therapy, and exercise [3,6]. According to a systematic review, cervical mobilisation and manipulation have comparable pain, function, and patient satisfaction benefits. A review has reported that several cervical manipulations over time may reduce pain and improve function compared with drugs and has also shown that cervical mobilisation (CM) is equally effective as manipulation [7]. Given the correlation between reduced thoracic spine mobility and neck pain and the increased risk of cervical manipulation, thoracic manipulation (TM) and CM were preferred in the control group in this study [8]. In addition, research has not sufficiently shown whether therapeutic exercises (TEs) benefit those with neck discomfort [9]. Although the plausible cause of cervicobrachial pain is mechanosensitive neural tissue, a study stated that only 19.9% of cases are neurogenic and implied that not all “positive tension tests” indicate negative neurodynamics [10,11].

According to previous studies, there are myofascial expansions (ME) where the deep fascia (DF) joins the various muscles of the upper quarter region (UQR), which may result in cervicobrachial discomfort and nociceptive pain [12]. The upper extremities, neck, and head are interconnected by the ME of the deep lamina of the deep cervical fascia (DCF), thus forming the myofascial continuum (MC) of the UQR [13]. The proprioceptors have the potential to develop into nociceptors, converting mechanical inputs into pain signals [12]. Also, an alteration in the hyaluronan’s viscosity leads to adhesions, resulting in the altered activation of the mechanoreceptors inducing pain [14,15,16,17]. Thus, dysfunctions of DCF may be a plausible causative factor of non-specific pain in the UQR associated with MNP. The restricted movement of collagen and elastin fibres due to the increased viscosity of the ground substance is restored by fascial manipulation (FM). The flexibility of myofascial structures may be increased by manipulating the points at the densified centre of coordination (CC) and centre of fusion (CF), which could improve fascial mobility and reduce symptoms [18,19]. Sequential yoga poses (SYP) restore fluid flow and improve the effectiveness and efficiency of the muscles by reducing the thixotropy of the ground substances [20,21,22]. The patient-specific functional scale (PSFS) and the numeric pain rating scale (NPRS), respectively, were employed in this study to evaluate function and pain [23].

The management of MNP usually demands an extensive course of treatment without a discernible benefit. Concomitant symptoms of the upper quarter region may develop because the DCF’s anatomical MC is impaired. This mandates a carefully planned study that uses DCF manipulation in MNP. Thus, this study intended to compare the effectiveness of FM of DCF and SYP to usual care, including home-based therapeutic exercises (TE), thoracic manipulation (TM), cervicothoracic manipulation (CTM), and cervical mobilisation (CM) in patients with subacute and chronic MNP.

## 2. Materials and Methods

### 2.1. Participants

In total, 125 participants aged 18–45 years with subacute or chronic MNP for three weeks or more were screened. Patients with inflammatory conditions, skin infections, bony lesions, vestibular balance issues, sensory or motor deficiencies of the upper quadrant, and UQR surgical/traumatic history within the past year were excluded from the study. The Institutional Research (IRC) and Ethics Committees (IEC) of Kasturba Medical College (KMC) and Kasturba Hospital (KH) (ECR/146/Inst/KA/2013/RR-19), MAHE, Manipal, Karnataka, India, gave their approval for the study. The trial was registered on 24 January 2020 at ClinicalTrials.gov (CTRI/2020/01/022934), with approval ID 790/2019 IEC.

### 2.2. Study Design

**Figure 1:**
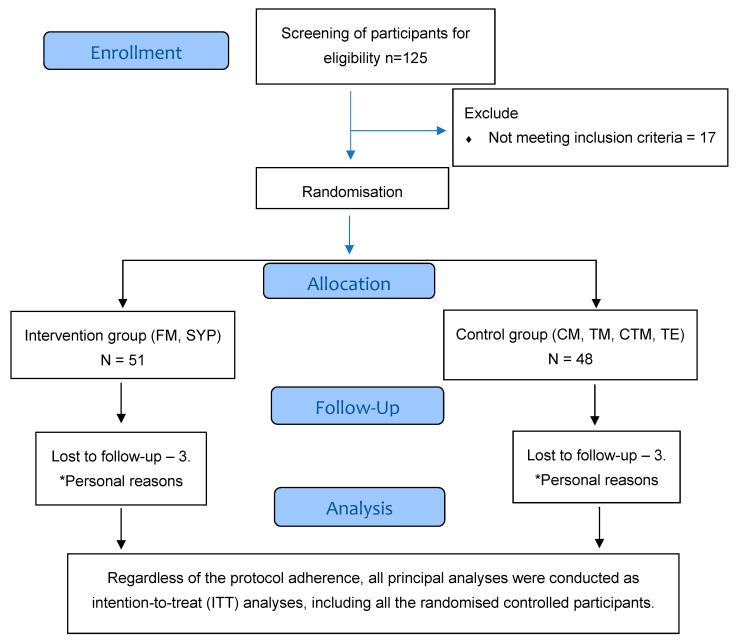
CONSORT diagram of the study design. * Indicating the reasons for withdrawal. FM—fascial manipulation, SYP—sequential yoga poses, CM—cervical mobilisation, TM—thoracic manipulation, CTM—cervicothoracic manipulation, TE—therapeutic exercise.

Two parallel groups were used in this pragmatic, outcome assessor-blinded, randomised controlled trial. Stratified (based on age) randomisation was implemented with an allocation ratio of 1:1. Ninety-nine participants were recruited for this study. The flow of this study is illustrated in Figure 1. 

### 2.3. Participant’s Recruitment

This study was conducted at the outpatient clinic of the Department of Orthopedics at Kasturba Hospitals, Centre for Sports Science, Medicine, and Research, Manipal Academy of Higher Education, Manipal and Lakshmi Memorial College of Physiotherapy, Mangalore, Karnataka.

### 2.4. Interventions

CM, TM, CTM, and TE were delivered to the patients in the usual care (control) group (CG), whereas the participants in the intervention group (IG) received FM. Both groups received treatment during day one, and the subsequent three treatment sessions had a minimum of 4 days between the sessions. Instructions about the home-based TE for the UCG and SYP for the IG were provided during the first treatment session and monitored during subsequent sessions. The concomitant use of NSAIDs, muscle relaxants, and analgesics was based on the physician’s recommendation and at the patient’s discretion. The medications used were recorded.

#### 2.4.1. Control Group (CM, CTM, TM, and TE)

Mobilisation includes oscillatory movements with a larger amplitude (for treating pain) and a smaller amplitude movement at the end of the range (for treating stiffness). For the treatment of muscle spasms, a sustained position was maintained at a point where the movement was restricted by muscle spasms and was interspersed with oscillatory mobilisation. Manipulation (unidirectional thrust movement) includes cervicothoracic and upper thoracic manipulation (rotation gliding C7–T3) and thoracic manipulation (rotation gliding T4–T9) [24,25,26,27,28].

Therapeutic exercises (TEs): home-based TEs, including upper cervical spine mobility and stretching of neck muscles, were taught. The craniocervical flexion (CCF) re-education and progressive training of deep neck flexors/extensors and axio-scapular muscles were also taught [3,8]. Muscle stretching of the suboccipital, levator scapulae, upper trapezius and sternocleidomastoid, was taught. The stretch position was held for 20–30 s and repeated 3–5 times daily. After re-educating the craniocervical movement, the training of the deep neck flexors and extensors was taught. Progressive retraining of the scapular control and training to improve the endurance of the synergistic muscles of the scapula was also taught [3]. TEs were performed following all four FM sessions under supervision. The participants were asked to perform unsupervised exercises at least five days a week from the beginning of the first treatment session until the final follow-up. Along with this, leaflets depicting the entire exercise regimen were provided. A standard protocol for the progression or regression of therapeutic exercises was based on pain response. In cases of a flare-up of symptoms lasting for more than a day, the intensity could be reduced, and the participants were able to correct the forms of these movements by looking at the instructions in the leaflet. The investigator gave adherence reminders during the initial and subsequent intervention sessions.

#### 2.4.2. Intervention Group (FM and SYP)

The intervention group received fascial manipulation^®^ (FM) and sequential yoga poses (SYP).

##### Fascial Manipulation^®^

Fascial manipulation^®^ (FM) includes locating the densified points, also known as the centre of coordination (CC) and centre of fusion (CF), on the deep fascia and applying manipulation (deep friction massage) for 5–8 min at each densified point using the knuckles or elbows [16,17,27]. High reliability was shown for the validity of movement and palpation verifications in coxarthrosis patients using the FM approach, even when carried out by inexperienced FM practitioners [27]. The exact locations of the CC and CF points and the treatment procedures are depicted in detail in the study protocol [10]. FM was performed by a physiotherapist with almost six years of experience providing this fascia-directed treatment approach.

##### Sequential Yoga Poses

**Figure 2:**
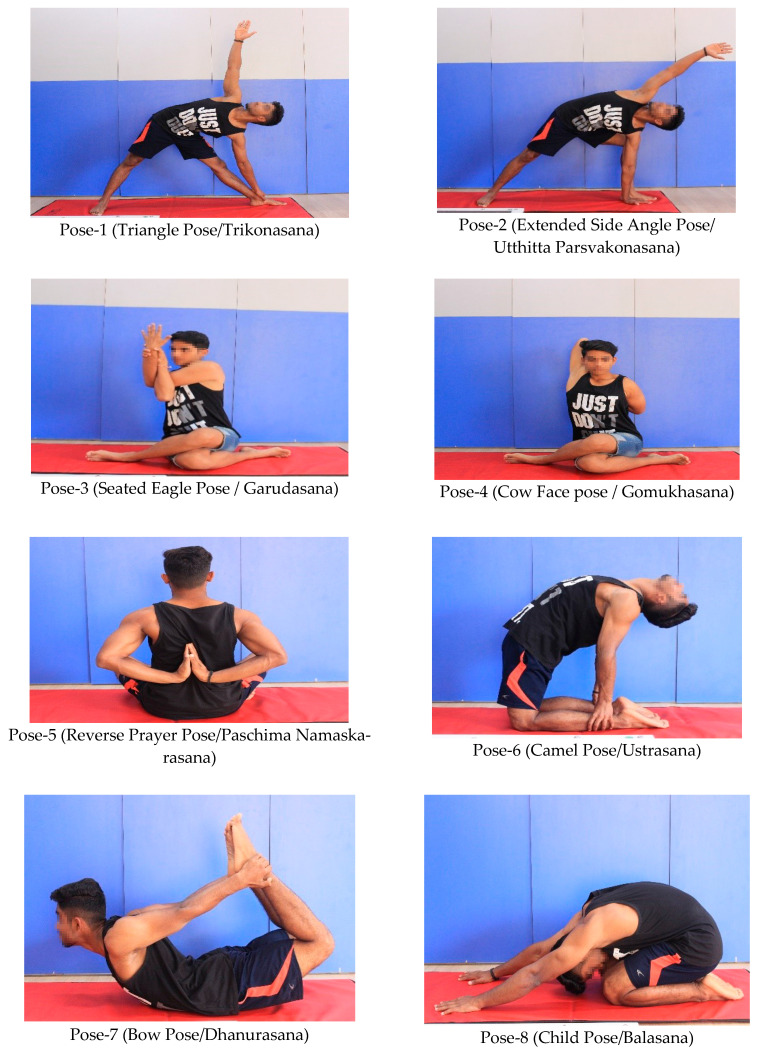
Sequential yoga poses.

The details of the sequences of SYP and the methods of poses are clearly illustrated in the study protocol [10]. SYP focuses on the myofascial continuum in the following sequence: the Triangle pose, Extended Side Angle pose, Seated Eagle pose, Cow Face pose, Child pose, Reverse Prayer pose, Camel Pose, Bow pose, and Child pose, as shown in Figure 2. Irrespective of the participants’ experience in Yoga, each pose was initially held for five breath cycles based on their ability to perform the pose. They progressed by increasing the number of breath cycles and the range in each pose [10,21]. Two repetitions of each pose were performed in the following order: Right → Left Right → Left.

The participants were instructed about the SYP, which is a home-based intervention performed by the participants on the first day of following FM. Supervised poses were performed following all four FM sessions. These poses’ form and progression were monitored during their follow-up visit. The participants were motivated to perform unsupervised Yoga poses for at least five days a week from the initial treatment session. Adherence in this study was improved by sending reminders and providing leaflets depicting the exercise regimen.

A standard protocol for the progression or regression of yoga poses was based on the pain response of the participants. In cases where a flare-up of symptoms occurred lasting more than a day, or if the participants found it challenging to perform the pose, the number of breaths in the hold position was reduced.

#### 2.4.3. Intervention Adherence

The effectiveness of rehabilitation exercises is increased by strict adherence to them. Few authors have reported that back pain patients obtain better benefits from strong exercise adherence [29,30]. The significance of the proper execution of TE and SYP was emphasised to the patients.

### 2.5. Outcomes

Primary outcome measures:

- Numeric pain rating scale (NPRS).

Secondary outcome measures:

- Patient-specific functional scale (PSFS)
- Fear-avoidance belief questionnaire—physical activity (FABQ-PA)
- Elbow extension range of motion during upper limb neurodynamics test 1 (ULNT1).

Numeric Pain Rating Scale

The NPRS is a valid and reliable scale used for MNP patients with moderate reliability (ICC = 0.67; [0.27 to 0.84]) [23]. A reduction of 2 points in the NPRS is usually regarded as the minimal clinically important difference (MCID) in patients with chronic musculoskeletal pain [31]. NPRS values were collected during the baseline and before the 2nd, 3rd, and 4th treatment sessions. Principal analysis was performed for changes in the mean between the groups from baseline to the 2nd, 3rd, and 4th treatment sessions.

Patient-Specific Functional Scale

In the PSFS, patients’ three most difficult activity limitations are quantified. The overall score is calculated by dividing the total number of activities by the sum of all activity scores. The minimum detectable change (MDC) is 2 points for average ratings and 3 points for single activity scores [32]. The data are shown as the mean difference between the groups. Analysis was conducted to identify changes between the baseline and the last treatment session as well as between the groups.

Fear-Avoidance Beliefs Questionnaire-Physical Activity (FABQ-PA)

The fear-avoidance beliefs questionnaire (FABQ-PA) examines patients’ dread of pain and subsequent avoidance of physical activity as a consequence of that fear [33]. FABQ-PA scores indicate more significant fear-avoidance beliefs above 15 [34]. FABQ-PA results contain a total score that can be considered elevated at 15 or higher. Lee et al. demonstrated that the FABQ-PA questionnaire is reliable, with an ICC value of 0.81 and a Cronbach’s alpha coefficient of 0.90 [35]. Principal analyses were performed to determine changes between the baseline and during the final treatment session, and the results are presented as the mean difference between the groups.

Elbow Extension ROM during the Upper Limb Neurodynamic Test

The elbow extension ROM (EEROM) is the region of the elbow range during ULNT1, where the patient feels discomfort measured with a universal goniometer [36].

This test was performed in the supine position, without a pillow, and with the head in a neutral rotation. The ULNT1 includes the following sequence of movements: shoulder girdle depression and then abduction to 90° with the elbow flexed at 90° followed by 70–90° of external rotation. The forearm was then supinated, followed by wrist and finger extension, and finally, elbow extension was performed. The elbow extension ROM was noted at the range where submaximal pain/discomfort or resistance prevented further movement [37].

The EEROM during ULNT1 correlates with the instant of submaximal pain in neck pain that can be quantified accurately in clinical settings [38]. The analysis was performed for changes from the baseline in every treatment session and between the groups.

### 2.6. Recruitment, Allocation, and Implementation

The patients underwent physical and radiographic assessments by the orthopedician. Patients with MNPs who had any alarming signs for manual therapy were excluded. Participants were randomly assigned to either the control or intervention groups using a 1:1 allocation ratio. The sequence was computer-generated using the www.randomiser.org website. Sixteen blocks of 10 people (5 in the CG and 5 in the IG) were used. The participants were divided into groups using sequentially numbered, sealed, and opaque envelopes. The participants who fulfilled the inclusion criteria and consented to participate in the trial were randomised. The outcome assessor was blinded, and the same outcome assessors performed all post-allocation assessments.

### 2.7. Statistical Methods

Repeated measures of ANOVA were used for all continuous primary and secondary outcomes. All statistical tests were performed at a 5% (two-sided) significance level using the IBM Statistical Package for Social Sciences 20 (IBM SPSS Statistics-20 for Windows, Armonk, NY, USA: IBM Corp.). The mean scores are reported for all the outcomes measured between different measurement time points. The differences in all outcomes between the interventional and control groups are reported. Regardless of the protocol’s adherence, all significant analyses—including all randomly assigned individuals—were carried out as intention-to-treat (ITT) analyses.

## 3. Results

### 3.1. Patient Characteristics

Three patients from the IG and three patients from the UCG withdrew during the follow-up period; as a result, 48 patients in the CG and 45 patients in the UCG completed the study. A post hoc power analysis was conducted using G* Power3 to test the difference between the two independent group means using a two-tailed test, an effect size of (d = 0.80), and an alpha of 0.05. The results showed that the power of the study was found to be 96% based on the total sample of 93, excluding the dropouts from both groups in this study [39]. The participant’s demographic characteristics are shown in Table 1.

### 3.2. Mean Values of the Outcomes during Subsequent Measurements

A repeated-measures ANOVA was performed to determine the effect of the IG (FM and SYP) compared to the CG (CM, TM, CTM, and TE) on NPRS and EEROM (over four measurement time points) and FABQ-PA and PSFS (over two measurement time points). The means and the standard deviations for all the dependent variables are reported in Table 2.

#### 3.2.1. NPRS

The Wilke lambda = 0.16, F (3, 90) = 156.98, *p* < 0.01, and partial η^2^ = 0.840, indicating a change in the NPRS score across the time frame with consecutive sessions. Additionally, there was a significant difference in the NPRS score with the group interaction, where Wilke Lamda = 0.699, F (3, 90) = 156.98, *p* < 0.01, and partial η^2^ = 0.301, indicating that the variation in the means of NPRS over repeated measurement occasions varied as a function of the group, as shown in Table 3.

Mauchly’s test indicates that the assumption of sphericity was violated, χ^2^ (5) = [32.483], *p* < 0.001; therefore, degrees of freedom were corrected using Huynh–Feldt estimates of sphericity (ε = 0.823). The within-subject effects on the NPRS score were statistically significant: Hyunh–Feldt F (2.469, 227.17) = 270.58, *p* < 0.001, partial η^2^ = 0.746. Additionally, the group had a significant interaction effect on NPRS, Hyunh–Feldt F (2.469, 227.17) = 22.48, *p* < 0.001. Partial η^2^ = 0.196, showing an interaction between the NPRS measurement occasions and the treatment groups. Additionally, the difference in the repeated measures of the NPRS over time differed between the treatment groups. The mean differences between the IG and CG in the NPRS 3rd and 4th sessions were −1.009 (*p* < 0.05) and −1.701 (*p* < 0.001), respectively, suggesting a significant reduction in pain in the IG when compared to the CG at the 3rd and 4th sessions, as depicted in Table 4.

#### 3.2.2. EEROM

The Wilke lambda = 0.58, F (3, 90) = 21.697, *p* < 0.001, and partial η^2^ = 0.420, indicating a change in the EEROM across the sessions. Additionally, there was a significant difference in the EEROM with the group interaction, Wilke Lamda = 0.744, F (3, 90) = 10.33, *p* < 0.01 and partial η^2^ = 0.256, indicating that the variation in the EEROM means during the subsequent measurements varied as a function of the group, as shown in Table 3.

Mauchly’s test (*p* < 0.001) implied that the sphericity assumption was not met, whereas the Greenhouse–Geisser Epsilon value was 0.491, suggesting the use of Greenhouse–Geisser adjustment. The within-subject effects on EEROM were statistically significant: Greenhouse–Geisser, F (1.472, 135.439) = 53.897, *p* < 0.001, and partial η^2^ = 0.369. Additionally, there was a significant treatment group interaction effect on EEROM: Greenhouse–Geisser F (1.472, 135.439) = 21.46, *p* < 0.001, and partial η^2^ = 0.189. These results showed an interaction between the EEROM measurement occasions and the treatment groups. Additionally, the differences in the repeated measures of EEROM over the subsequent sessions differed across the treatment groups. These results are presented in Table 3.

The Bonferroni-adjusted pairwise comparison of each group’s average EEROM between the sessions and within subjects indicates a statistically significant difference (*p* < 0.001). There were no differences between the IG and CG between the baseline and first three sessions. The mean difference between the IG and CG in EEROM in the fourth session was 20.120 (*p* < 0.001), suggesting a considerable improvement in EEROM in the IG compared to the CG from baseline to the fourth session, as shown in Table 4.

#### 3.2.3. FABQ-PA

The Wilke lambda = 0.565, F (1, 91) = 70.023, *p* < 0.01, and partial η^2^ = 0.435, indicating a change in the FABQ-PA in the fourth session compared to the baseline. Additionally, there was a significant difference in the FABQ-PA score with the group interaction, Wilke Lamda = 0.921, F (1, 91) = 7.806, *p* = 0.06, and partial η^2^ = 0.079, indicating that the variation in the means of FABQ-PA measurements on different occasions varies as a function of the group. Mauchly’s test of sphericity (*p* < 0.001) indicated that the assumption of sphericity was not met, whereas the Greenhouse–Geisser Epsilon value was >0.75, suggesting the use of the Huynh–Feldt adjustment. The within-subject effects on FABQ-PA were statistically significant with Huynh–Feldt F (1, 91) = 70.023 and *p* < 0.001. Additionally, there was a significant difference in the between-group interaction effect with Huynh–Feldt F (1, 91) = 7.806 and *p* = 0.006. As reported in Table 3, these results demonstrated an interaction between the FABQ-PA measurement occasions and the treatment groups. Additionally, the differences in the measurement of EEROM from the baseline to follow-up differed across the treatment groups. The mean difference between the IG and CG of the FABQ-PA during the follow-up was −5.036 (*p* < 0.001), indicating a statistically significant difference in the FABQ-PA during the follow-up compared to the baseline score and is reported in Table 4.

#### 3.2.4. PSFS

The Wilke lambda was statistically significant, Wilke lambda = 0.535, F (1, 91) = 79.023, and *p* < 0.01, indicating a change in the PSFS from baseline to follow-up. Additionally, there was a significant difference in the PSFS with the group interaction, Wilke Lamda = 0.953, F (1, 91) = 4.517 and *p* = 0.36 indicating that the variation in the means of PSFS measurement during the follow-up varies as a function of the group.

Mauchly’s test (*p* < 0.001) indicated that the sphericity assumption was not met. The Greenhouse–Geisser Epsilon value was >0.75, suggesting the use of Huynh–Feldt adjustment with the univariate test of the mean difference. The within-subject effects on PSFS were statistically significant: Huynh–Feldt F (1, 91) = 79.043, *p* < 0.001. Additionally, there was a substantial difference in the between-group interaction effect with Huynh–Feldt F (1, 91) = 4.517, and *p* < 0.001, as indicated in Table 3.

**Figure 3:**
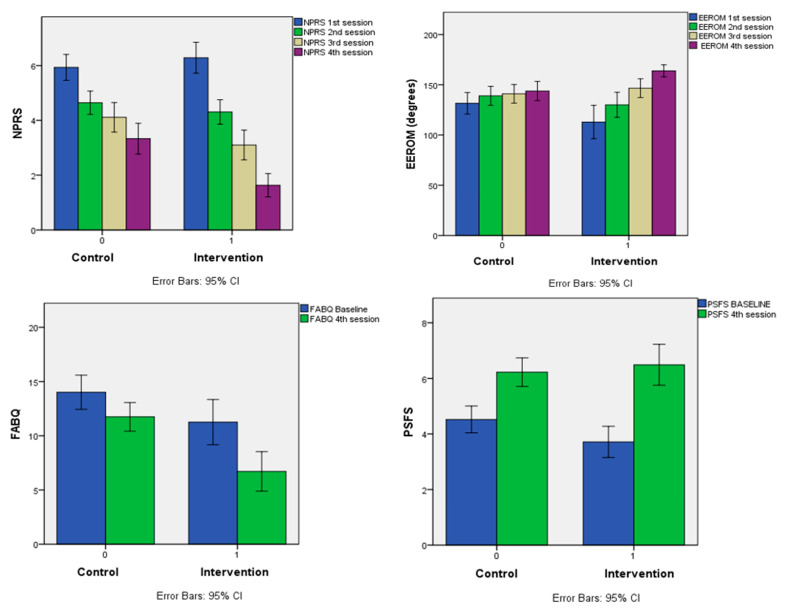
Comparison between the CG and IG effects on the outcomes at different measurement time points. NPRS—numerical pain rating scale, EEROM—elbow extension range of motion, FABQ—Fear-Avoidance Behavior Questionnaire, PSFS—patient-specific functional scale, CG—control group, IG—intervention group.

The Bonferroni-adjusted pairwise comparison of each group’s average PSFS scores (averaged across the sessions) was not statistically significant, *p* = 0.412. The mean difference in PSFS between the IG and CG during follow-up was 0.263 (*p* = 0.566), suggesting no difference in the PSFS value in the IG compared to the CG, as specified in Table 4. The comparison of effects between the CG and IG on all the outcomes at different time points of measurement is shown in Figure 3.

## 4. Discussion

MNP is a complex musculoskeletal painful disorder where pain is a disabling component, and it can eventually cause severe dysfunctions and significant challenges with daily living activities. We assessed pain, function, and ROM as outcomes related to treating MNP. The FM targeting the densified points on the myofascial continuum of the upper quadrant and the Yoga poses focusing on the entire MC represent the treatment strategies that can improve pain, function, and ROM [10,40]. Thus, FM was associated with SYP to evaluate their efficacy in managing MNP in this study. The results indicate that there was a reduction in pain and fear-avoidance beliefs (FAB), as well as improvement in function and EEROM, during the subsequent treatment sessions in both the IG (FM and SYP) and CG (CM, TM, CTM, and TE). The results demonstrate a significant reduction in pain (third and fourth sessions) and FAB, as well as an improvement in function and EEROM (fourth session) in the IG compared to the CG.

The reduction in pain, as measured by the NPRS, was statistically significant. The minimal clinically important difference (MCID) for chronic persistent musculoskeletal pain was considered to be 2 (i.e., patients are usually considered improved when there is a reduction in NPRS by 2) [31]. Few authors have reported that the cut-off values for the change in NPRS for the moderate (4–6), severe pain (7–10), and moderate plus severe pain (4–10) groups were 1.3, 1.8, and 1.5, respectively, based on the pretreatment pain score. Therefore, it was suggested that, for practical purposes, MCID values for changes in the NPRS scale can be rounded up to 1.5 regardless of the degree of pain severity prior to the treatment [41]. Given this, there was a noticeable decrease in pain in the third and fourth sessions in the IG compared to the CG. Considering the partial eta squared (η^2^*_p_*) values of 0.07 (third session) and 0.207 (final session), there were moderate and large effect sizes in the third and fourth sessions, respectively, between the IG and CG. This study’s findings are consistent with those of a recent study, where subsequent therapy sessions saw a significant reduction in pain using standard FM. By contrast, the modified FM method showed a substantial pain reduction in the third session and 1-month follow-up [42].

The EEROM during the ULNT1 data indicated a substantial improvement in the EEROM in all subsequent sessions compared to the previous sessions in both the intervention and control groups. Few studies have reported that an improvement of approximately 7.5° in EEROM can be considered a minimally important difference [36,38]. In this study, the mean difference between the IG and CG in EEROM in the fourth session was 20°, suggesting a considerable improvement in EEROM in the IG compared to the CG from the baseline to the fourth session. Additionally, the η^2^*_p_* value of 0.126 in the last session showed a larger effect size in the EEROM during ULNT1 when the IG was compared with the CG. Costello M et al. reported greater immediate improvements in EEROM during ULNT1 following soft tissue mobilisation in patients with cervicobrachial pain [36]. Patients with reduced neural extensibility in their upper limbs, as indicated by decreased EEROM during ULNT1, showed a decreased length of the upper trapezius, thus showing that restrictions in the soft tissues surrounding the nerves may impair neural mobility [43]. Few authors have discussed the plausible involvement of deep fascia in fascial entrapment neuropathy, which presents a complex diagnostic problem [44]. Considering the rich innervation of the deep fascia and the presence of tender taut bands on the soft tissues of the UQR associated with myofascial dysfunctions may also give nociceptive input to the nervous system, thus contributing to the MNP perceived by the patient [45,46]. Thus, in this study, although the treatment in the IG targets the soft tissues of the UQR, there is a profound improvement in neural extensibility, as indicated by an increase in the EEROM during ULNT1.

The mean difference between the IG and CG on the FABQ during the follow-up was −5.036 (*p* < 0.001), suggesting a statistically significant difference in FABQ scores in the IG compared to the CG. A change of four points on the FABQ-PA is considered MCID, which seems to identify meaningful changes in fear-avoidance beliefs [47] accurately. There was a reduction of approximately 5 points in the IG compared to the change of just 2 points in the CG. Additionally, the IG had a larger effect size (η^2^*_p_* = 0.17) during the fourth session compared to the baseline. Thus, the IG can be considered clinically significant in reducing the FAB compared with the CG in this study.

PSFS data showed a change in the PSFS value from the baseline to follow-up in both the IG and CG. The mean difference in PSFS between the IG and CG during follow-up was 0.263 (*p* = 0.566), suggesting no statistically significant difference. Similarly, there was a very small effect size (η^2^*_p_* = 0.04) when comparing the fourth session with the baseline between the control and intervention groups. However, a study reported that the minimum detectable change (MDC) for PSFS is two points for average ratings [32]. The mean change in the PSFS score from the baseline (4.52 ± 1.592) to follow-up (6.23 ± 1.696) in the CG was <2, whereas the mean change from the baseline (3.71 ± 1.958) to follow-up (6.49 ± 2.55) in the IG was >2, which is above the MDC, thus clinically showing that the IG may be better at improving the PSFS than the CG. The positive influence of FM on restoring function was also reported in a recent study by Kamani et al. in patients with chronic ankle instability [19].

The reduction in pain and improvement of function and ROM in the IG compared to the CG can be attributed to the FM, where the manual friction provided during FM restores tissue gliding where there is increased stiffness [40,42].

## 5. Conclusions

Although there was a substantial reduction in pain and fear-avoidance beliefs (FAB), as well as an improvement in function and EEROM during the subsequent treatment sessions in both the IG and CG, there was a significant reduction in pain and FAB during the third and fourth session as well as an improvement in function and EEROM during the final follow-up in the IG comparing to the CG. As MNP contributes significantly to overall disability and considerable societal burden, the fascia-directed approaches, such as FM and SYP, that focus on the entire myofascial sequences can be considered valuable in effectively managing patients with MNP. By reducing pain and fear-avoidance behaviour as well as improving the function and extensibility of the upper quarter region, the FM and SYP can be incorporated to improve the functional outcomes in non-specific neck pain and dysfunctions. A proper long-term evaluation of the treatment’s effects was not possible due to the short (30-day) follow-up time. Thus, future studies are needed to investigate the long-term efficacy of FM and SYP.

## Figures and Tables

**Table 1:** Participants’ demographic characteristics.

Characteristics	Group		Mean ± SD
Age	CG	N = 48	26.1 ± 5.83
	IG	N = 51	27.8 ± 8.00
Gender: n (%)	CG	Male	27 (27.3)
		Female	21 (21.2)
	IG	Male	23 (23.2)
		Female	28 (28.3)

N—number of participants, FM—fascial manipulation, SYP—sequential yoga poses, CM—cervical mobilisation, TM—thoracic manipulation, CTM—cervicothoracic manipulation, TE—therapeutic exercises, SD—standard deviation.

**Table 2:** Mean values of outcomes during different time points of measurement.

Timeline	Group	NPRS (Mean ± SD)	EEROM (Mean ± SD)	FABQ (Mean ± SD)	PSFS (Mean ± SD)
Baseline	CG	5.93 ± 1.572	131.60 ± 35.70	14.02 ± 5.187	4.52 ± 1.592
	IG	6.29 ± 1.969	112.92 ± 57.960	11.27 ± 7.274	3.71 ± 1.958
2nd session	CG	4.64 ± 1.417	139.02 ± 31.484		
	IG	4.31 ± 1.557	130.04 ± 43.561		
3rd session	CG	4.11 ± 1.799	141.02 ± 30.881		
	IG	3.10 ± 1.896	146.61 ± 32.495		
4th session	CG	3.33 ± 1.871	143.78 ± 31.947	11.75 ± 4.352	6.23 ± 1.696
	IG	1.63 ± 1.482	163.90 ± 21.045	6.71 ± 6.334	6.49 ± 2.559

NPRS—numerical pain rating scale, EEROM—elbow extension range of motion, FABQ—Fear-Avoidance Behaviour Questionnaire, PSFS—patient-specific functional scale, CG—control group, IG—intervention group.

**Table 3:** Group interactions with all outcomes at different measurement time points.

	Outcomes	F Value	*p*-Value	Partial η^2^
Huynh–Feldt	NPRS	270.58	<0.001	0.746
	NPRS * Group	22.482	<0.001	0.196
Greenhouse–Geisser	EEROM	53.89	<0.001	0.369
	EEROM * Group	21.46	<0.001	0.189
Huynh–Feldt	FABQ	70.02	<0.001	0.435
	FABQ * GROUP	7.806	0.006	0.079
Huynh–Feldt	PSFS	79.04	<0.001	0.465
	PSFS * GROUP	4.517	0.036	0.047

* Interaction of group with the outcome, η^2^—eta squared, NPRS–numerical pain rating scale, EEROM–elbow extension range of motion, FABQ–fear-avoidance behaviour questionnaire, PSFS–patient-specific functional scale,

**Table 4:** Mean differences between IG and CG of all outcomes.

Timeline	NPRS				EEROM				FABQ				PSFS			
	MD	SE	*p*-Value	η^2^*_p_*	MD	SE	*p*-Value	η^2^*_p_*	MD	SE	*p*-Value	η^2^*_p_*	MD	SE	*p*-Value	η^2^*_p_*
Baseline	0.352	0.370	0.343	0.010	−18.6	10.03	0.066	0.036	−2.75	1.32	0.04	0.046	−0.80	0.37	0.033	0.049
2nd session	−0.338	0.308	0.275	0.013	−8.98	7.90	0.259	0.014								
3rd session	−10.00	0.382	0.010	0.070	5.59	6.55	0.396	0.008								
4th session	−10.70	0.347	<0.001	0.207	20.12	5.53	<0.001	0.126	−5.03	1.14	<0.001	0.17	0.26	0.45	0.566	0.04

η^2^*_p_*—partial eta squared MD-mean difference, SE—standard error, NPRS—numerical pain rating scale, EEROM—elbow extension range of motion, FABQ—Fear-Avoidance Behaviour Questionnaire, PSFS—patient-specific functional scale, CG—control group, IG—intervention group.

## Data Availability

On request, the corresponding author will provide access to data used in this work. Due to ethical constraints, data are not publicly accessible.
